# Early Lessons From Ethiopia in Establishing a Data Triangulation Process to Analyze Immunization Program and Supply Data for Decision Making

**DOI:** 10.9745/GHSP-D-21-00719

**Published:** 2022-06-29

**Authors:** Adriana Almiñana, Amare Bayeh, Daniel Girma, Natasha Kanagat, Lisa Oot, Wendy Prosser, Belayneh Dagnew, Disha Ali

**Affiliations:** a JSI Research & Training Institute, Inc., Arlington, VA, USA.; b JSI Research & Training Institute, Inc., Addis Ababa, Ethiopia.

## Abstract

Strengthening data use and quality is critical to achieving high, equitable immunization coverage. One approach that is being increasingly recognized as effective in improving data use and quality is data triangulation, which can provide more information for decision making in public health programs.

In Ethiopia, immunization program data has had ongoing quality challenges, including timeliness, completeness, and accuracy. Some data are reported through different systems to different departments, and coordination between departments is limited.

JSI, through the Universal Immunization through Improving Family Health Services (UI-FHS) project, introduced a data review process and an Excel tool for triangulating immunization program data and vaccine supply data to improve data quality and programmatic decision making. The user-friendly Immunization Data Triangulation Tool (IDTT) provides decision-support information—such as scoring of districts based on performance—and suggests follow-up actions. It also highlights gaps between vaccines supplied and consumed and helps managers determine the next steps to address programmatic, supply, or data quality issues.

The data review process and IDTT were rolled out in 2 regions in Ethiopia. UI-FHS documented learning to understand the feasibility of the IDTT's application as a decision-making tool by conducting key informant interviews and observing how the IDTT was used at monthly data review meetings.

Health managers who used the tool reported ease of use and clear benefits, including more accessible and synthesized data, which prompted decision making and actions to improve services and supply, such as expanding the number of immunization sites. Challenges with the availability of vaccine supply data hindered managers' ability to leverage triangulated data fully, but the data triangulation process prompted cross-departmental collaboration to address this gap.

These early findings show promise in the ability of immunization programs to successfully use triangulated data to address challenges and provide lessons for introducing new tools or processes into health systems.

## Abstract (precis)

Health managers in Ethiopia used a data triangulation tool and process to prompt decision making and meaningful actions to improve immunization services. Immunization managers interested in incorporating data triangulation analyses into their current data review systems will need to determine practical, feasible means for operationalization, preferably through a collaborative, iterative process with users.

## Teaser

**Key Findings:**

- Health managers who reviewed data by using the data triangulation tool reported clear benefits, including more accessible and synthesized data. This prompted decision making and meaningful actions to improve immunization services, such as expanding the number of immunization sites.
- Additional effort was required initially to improve the availability of vaccine supply data to fully leverage triangulated data. Over time, health managers could operate the tool on their own and lead the meeting and data review process.

**Key Implications:**

- More research and documentation are needed to further understand what regular, practical data triangulation processes can look like for Expanded Programmes on Immunization (EPIs) and how data triangulation processes contribute to better programmatic decision making and better data quality.
- EPI managers interested in incorporating data triangulation analyses into their current data review systems will need to determine practical, feasible means to operationalize regular use of triangulated data at the subnational level. When doing so, the process should be designed through a collaborative process with users, including an initial training followed by iterative support on a routine basis, embedding of implementation research, and a collaborative process to work with users to address barriers or challenges.

## BACKGROUND

Strengthening data use and quality is critical to achieving high, equitable immunization coverage, both globally and in Ethiopia, which ranks among the top 10 countries with the most under- and unvaccinated children.^1^ Streamlining data systems, promoting evidence-based decision making, improving data visibility, and strengthening feedback systems are all efforts that have been noted as key to progressing on global and country-level strategies, including Gavi's 5.0 strategy,^2^ the World Health Organization's (WHO) Immunization Agenda 2030,^3^ and Ethiopia's Health Sector Transformation Plan.^4^

Data triangulation—the synthesis of 2 or more existing data sources to address relevant questions for program planning and decision making^5^—is being increasingly recognized as a means to improve data use and quality in public health programs, including immunization, and to improve program decisions. Evidence has shown that data quality can improve through programs actually using data,^6^ so by triangulating several pieces of data, even if they are imperfect, decision makers can gain an additional layer of insight and understanding to improve their basis for decision making.^5^ One common way to triangulate data is by comparing health program data with product/commodity supply data. For example, in Laos, data on rates of malaria cases and testing is compared with stock data to understand what health facilities' needs are for malaria commodities.^7^ While there are many potential ways to triangulate immunization data, documentation is limited on the specific techniques or processes undertaken by ministries of health.

By triangulating several pieces of data, even if they are imperfect, decision makers can gain an additional layer of insight and understanding to improve their basis for decision making.

In Ethiopia, administrative data for immunization—the data collected and reported through the government health system—has ongoing quality challenges, including timeliness, completeness, and accuracy.^8^^,^^9^ For example, administrative data can differ considerably from survey estimates of immunization coverage, with administrative data often skewing higher (e.g., administrative coverage for the third dose of the pentavalent vaccine in 2019 was 96%, while the WHO/UNICEF estimate was 69%).^10^ In addition, some indicators relevant to programmatic decision making are reported through different systems to different responsible departments, such as immunization coverage data that are reported up through District Health Information System 2 (DHIS2) and vaccine supply related indicators, which are reported through a logistics management data system called mBrana. Coordination is limited between program staff at the Expanded Programme on Immunization (EPI) within the Ministry of Health (MOH), who generally focus on DHIS2 data, and supply chain staff within the Ethiopian Pharmaceutical Supply Agency (EPSA), who generally focus on mBrana data. Limited coordination has meant that regional EPI officers do not consistently incorporate supply chain data into their decision-making processes, and review or use of data overall for program improvement is not always consistent.

JSI, through the Universal Immunization through Improving Family Health Services (UI-FHS) project, supported the MOH over the past decade to improve the EPI, including providing technical assistance to 5 of Ethiopia's 11 regional health bureaus (RHBs) since 2018. The RHB in Benshangul Gumuz (BG) region requested support in 2019 to improve the use of immunization program and supply data for programming and decision making. We describe JSI's experience and lessons learned from introducing a process and tool for triangulating immunization program and supply data for use at the regional level to support regular feedback and action at the district level. It contributes to the literature on and practical experiences with triangulating data for data use and decision making.

## DEVELOPMENT OF DATA TRIANGULATION TOOL AND DATA REVIEW PROCESS

JSI held a consultative workshop with BG RHB stakeholders to develop a practical approach and structured process for using and analyzing triangulated program and supply data on a regular basis. The group discussed specific indicators that could be compared or analyzed together to provide greater insight into program performance, at what level this data triangulation process should occur (at the regional level on a monthly basis to bring together cross-departmental staff who could take feedback to the districts), what review processes should look like, and how to merge and analyze the data. Since time and technical capacity to integrate disparate datasets were limited, stakeholders determined that developing a tool to automate analysis would be critical to facilitating regular review of triangulated data.

In collaboration with RHB stakeholders, JSI codeveloped a Microsoft Excel-based Immunization Data Triangulation Tool (IDTT) (Supplement 1). The tool was designed to triangulate service statistics indicators from DHIS2 and supply chain indicators from mBrana, the logistics management data system to provide a comprehensive picture of immunization program and supply chain data in a user-friendly way. Several indicators were compared against each other to produce ratios that provide insight into data quality issues between stock delivered and consumed or programmatic issues such as consistent understock; more detail on the indicators used is included in Supplement 2. Coverage for all antigens was also included in the IDTT, as well as WHO Reaching Every District categorization scores, which classify performance based on pentavalent vaccine coverage and dropout.^11^ An IDTT user guide (Supplement 3) was also developed, which provided information on the rationale and process for conducting data triangulation, provided guidance on the data review process, and instructed users in a step-by-step manner on the use of the IDTT Excel tool.

The Immunization Data Triangulation Tool was codesigned with RHB stakeholders to provide a comprehensive picture of immunization program and supply chain data.

Using color coding, IDTT dashboards highlighted districts that require programmatic attention and those that are performing well. The IDTT provided decision-support features, such as scoring based on performance and suggested actions managers should take based on scores. The tool was thus meant to trigger actions by managers across departments to support changes at the district level, such as examining discrepancies between vaccine doses supplied and administered. The Figure outlines the structured data review process that was used to facilitate data use, provide a collaborative forum through which staff from multiple departments could identify action points, and track improvements and potential outcomes based on action taken.

**FIGURE:**
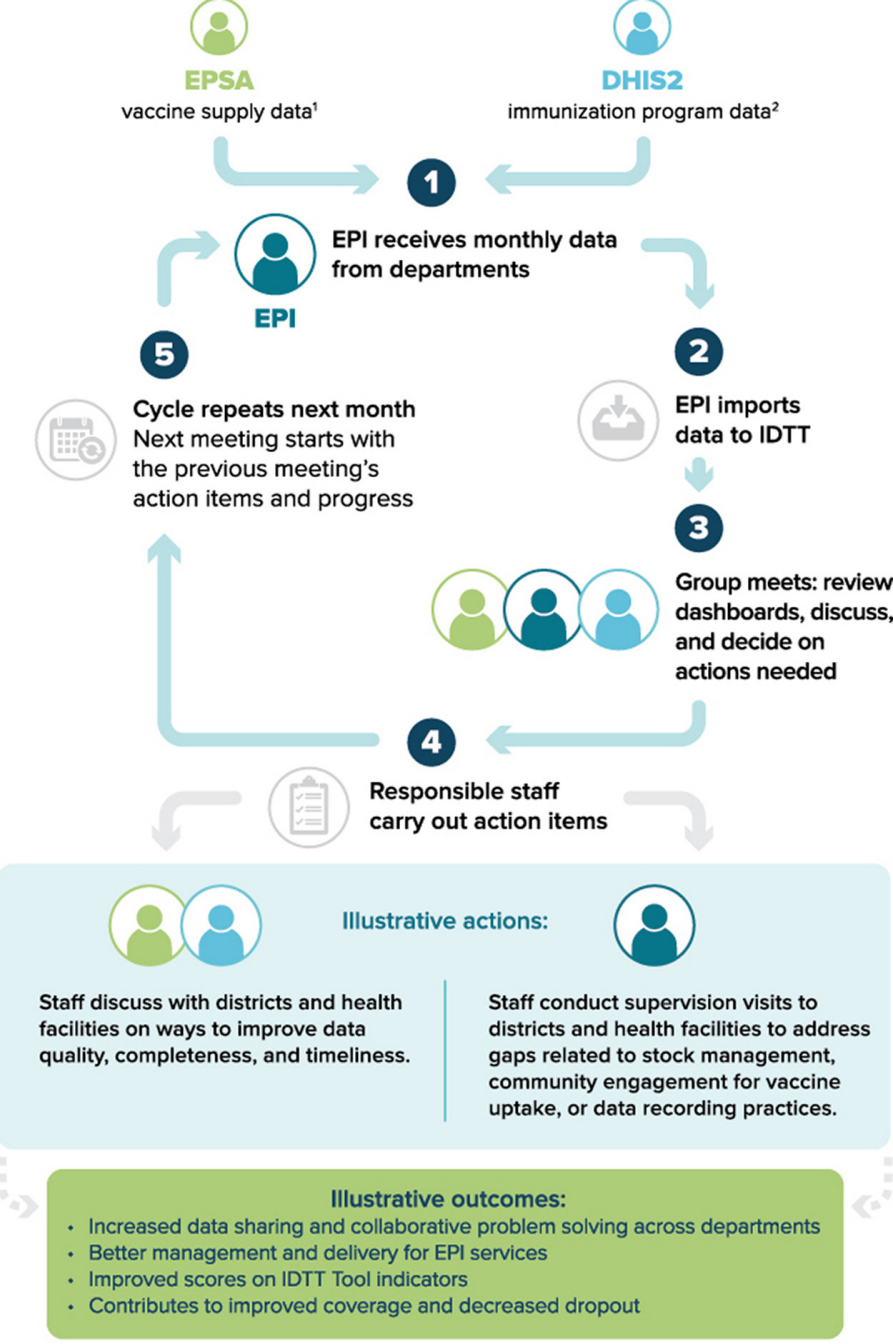
Conceptual Framework for Data Triangulation Data Review Process in Ethiopia, Including Illustrative Actions and Outcomes Abbreviations: DHIS2, District Health Information System 2; EPI, Expanded Programme on Immunization; EPSA, Ethiopian Pharmaceutical Supply Agency; IDTT, Immunization Data Triangulation Tool. ^1^ Stock on hand and vaccine issue data from mBrana logistics management system. ^2^ Immunization coverage data from DHIS2.

JSI rolled out the IDTT and data review process in 2 management units: at the RHB level in BG region, and the zonal health department (ZHD) level (a management level between regional and district) in Kembata Tembaro (KT) zone within the Southern Nations, Nationalities, and Peoples Region (SNNPR). Both were within the project's overall scope geographically, and they were chosen to implement this activity because of their expressed interest and the desire to test the tool/process at 2 different management levels. Roll-out included several activities. Upon finalizing the tool's development, JSI provided an initial in-depth orientation and distributed the IDTT user guide. Then, ongoing follow-up support was provided throughout implementation, eliciting users' feedback on the tool through individual and group discussions and helping facilitate monthly data review meetings led by the RHB and ZHD. Monthly meetings took place from October 2020 to May 2021 for KT ZHD and March to May 2021 for BG RHB.

## PROCESS DOCUMENTATION OBJECTIVE AND METHODS

The process documentation aimed to understand the feasibility of the IDTT's application as a decision-making tool by examining its use during review meetings. The learning particularly focused on examining the IDTT's use in cross-departmental problem solving, the decisions made based on the data analysis, the actions taken (e.g., supportive supervision conducted), and the outcomes of those actions (e.g., improved availability or quality of data). This information would be used to share lessons learned on if and how the IDTT tool and process could address problems within the immunization program.

The process documentation applied mainly qualitative methods to understand the feasibility, benefits, and challenges regarding the data triangulation process and use of the tool. We conducted key informant interviews (KIIs) with representatives of the RHB and ZHD, EPSA, health management information system, and JSI project staff involved in this activity. A team of experienced local researchers conducted 14 KIIs (7 in each region, BG and SNNPR) in Amharic using semistructured open-ended guides. KIIs were transcribed and translated into English, and a simple text analysis was conducted following a list of themes and codes aligned with the study's objective. In addition, we examined the data uploaded in the IDTT and meeting minutes over the period of implementation to track the data being uploaded and the decisions that were being made as a result of data review.

### Ethical Approval

We obtained ethical clearance from JSI's institutional review board and additional clearances to conduct KIIs from both regions' RHBs. Before the interviews started, all participants gave their verbal consent.

## FINDINGS

### 1. Perceptions of Early-Stage Effectiveness

While the early stage of introduction differed in each region, stakeholders from both regions recognized the value of using triangulated program and supply data.

The KT ZHD demonstrated faster uptake of the tool and approach, leading to more regular use of the tool for decision making, consistent data review meetings, and increasing ease of using the tool. Members attending the triangulation data review meeting on a monthly basis included EPI staff; broader maternal, newborn, and child health (MNCH) staff; and logistics staff (who communicate with regional-level EPSA staff). Respondents reported that during IDTT data review meetings, committee members interacted and examined the data holistically. For example, logistics staff responsible for reviewing immunization supply and stock data communicated directly with EPI staff, whose usual concerns were coverage data from DHIS2.

The KT ZHD demonstrated faster uptake of the tool and approach, leading to more regular use of the tool for decision making, consistent data review meetings, and increasing ease of using the tool.

Respondents reported that simultaneous review of immunization coverage and supply data was a new process; previously, the process had focused mainly on reviewing coverage data. Based on the decisions made in the data review meetings, follow-ups for the action items were conducted during supportive supervision and/or by directly communicating with districts, and updates were shared in subsequent data review meetings.

Committee members also reported increased comfort and ease with using the tool—such as mastering data import, navigating dashboards, and understanding the outputs from analyses—as they continued to use it over time.

The BG RHB experienced a slower roll-out. Since the process documentation exercise occurred 3 months after the IDTT tool was rolled out, the activity in BG was still in a nascent stage. Despite the slow start, respondents reported beginning to populate the tool and meeting as a committee. Some key discussion points focused on missing supply data (region-wide issue) and missing program data (from districts experiencing security issues). For the districts experiencing security issues and missed reporting, meeting minutes showed the intention to develop plans based on the affected districts' needs. However, due to the coronavirus disease (COVID-19) and ongoing security threats, these plans had not yet been executed. Although full use of the tool was constrained by lack of stock data, respondents expressed interest in continuing to use the IDTT with improved supply data.

Many respondents in both regions reported clear benefits to using the tool and data review process, including respondents in BG who had only recently initiated the activity. Respondents from both regions noted the following benefits.

- The tool allowed for easy comparison and analysis of data from different sources in a single platform; data were more accessible, and the tool eased data management for decision making, thereby reducing workload.
- Synthesis of data and information helped identify districts that were functioning better and those that were not, and regular updating of the tool helped monitor progress over time.
- The tool and data review process helped improve and maintain data quality, which is an important policy priority for the MOH.
- Color coding used in the tool's dashboards helped visualize data and eased interpretation of data.

*The trend of triangulating supply chain data and the service data was not known previously. We can now monitor monthly improvements in the immunization program and take immediate actions to address the gaps. Outbreaks have reduced in our zone*. —SNNPR Public Health Emergency Management representative

### 2. Barriers to Effective Implementation During Roll-Out

BG experienced greater challenges with introducing the tool. EPSA, a critical partner responsible for ensuring the timely supply of vaccines, was unable to participate due to competing priorities. Recurring conflict/ethnic clashes and competing priorities such as COVID-19, limited the RHB staff's availability to engage in IDTT implementation and affected the availability of data. These issues affected the functionality and performance of the region's immunization program as a whole and caused a delay in the data triangulation activity's implementation, which started in March 2021.

In both regions, mBrana often had incomplete supply chain data, which led staff to focus more on indicators/analysis based on program data, while at the same time raising the issue of incomplete or missing vaccine supply data for discussion and action at higher levels. KIIs indicated that committee members discussed data gaps and developed action plans collaboratively.

*We download the DHIS2 [and mBrana] data, and then one of us displays the results over a projector… We then discuss each finding to identify if there is low coverage or dropout rates for each woreda (district). We then provide feedback to the woredas based on findings; if good, we just write feedback to keep up the good deeds; or if poor performance, we provide action points for improvement depending on the problem.* — EPI focal person

In both regions, mBrana often had incomplete supply chain data, which led staff to focus more on indicators/analysis based on program data.

Respondents from BG RHB reported more challenges than respondents from KT ZHD. This aligns with the finding that familiarity and comfort with the tool increased over time and the fact that the activity was still in an early stage in BG. Overall, respondents reported indicator- or analysis-specific challenges or operational hurdles. Respondents reported some of the following challenges.

- Some respondents desired additional indicators (e.g., raw numbers of unimmunized children in addition to percent coverage) or for indicators to be summarized in a different manner (e.g., quarterly summary in addition to monthly analysis).
- The tool was perceived as somewhat complex; interpreting output values takes time and practice to understand.
- The tool requires some knowledge of Microsoft Excel and computer skills.

### 3. Demonstrated Potential for Impact

One important recognition that came out of the early stages of introduction was the importance of having data available, particularly supply data. Before commencing the activity in BG, the RHB and EPSA took steps to improve data completeness in mBrana, and to some extent in DHIS2, as reporting to these systems had lapsed considerably in the previous 6 months due to conflict and other issues. This initial step was needed to ensure adequate data were available to begin to triangulate and analyze. While implementing the IDTT and data review process in both regions, improving the availability of vaccine supply data continued to be a focus to strengthen data systems.

One documented outcome was an expansion of outreach (community-based) immunization sites. By using the IDTT tool, the KT ZHD team identified lower-than-expected coverage in a town despite adequate vaccine supply. The team communicated with EPI officers in the town and identified the reasons, which were poor planning and a shortage of outreach sites. As a result, the ZHD, in collaboration with the district health office, decided to launch new outreach sites.

One documented outcome of using the IDTT was launching new outreach immunization sites in KT to address low coverage in a town.

Another documented outcome was the redefining of a target population. The data review process revealed that a small town in a more urban area demonstrated poorer performance compared to its neighboring communities. The KT ZHD team investigated the cause and found that some neighboring rural areas with low coverage had been recently rezoned into the town, which resulted in a high number of unimmunized children. The EPI team took immediate measures and adjusted its plan to redefine the target population.

## LESSONS LEARNED

This experience provided lessons for establishing a systematic and recurring process to use triangulated data in immunization and for introducing tools and processes into health systems.

### Establishing Regular Processes for Using Triangulated Data in Immunization

1. **Triangulation can foster better coordination among health staff with different professional roles:** This is important to analyze data from different perspectives and encourage group problem solving.
2. **Regular availability of data is important to fully leverage the benefits of triangulation:** To optimally triangulate data, it is essential to ensure regular reporting of data to the data systems that will be used for triangulation analysis; otherwise, the indicator measures could fall short of achieving desired objectives. Efforts to improve and sustain data availability/completeness should be prioritized.
3. **Subnational managers could use data triangulation to monitor issues at the district level:** Given that data triangulation can be complex, having subnational managers use data triangulation as a management tool at the regional level to address problems happening at the district level showed some success. This takes ongoing practice and support.

### Introducing Tools and Processes Into Health Systems

1. **Design with and for the users:** The stakeholders were engaged from the beginning to codesign a tool with features that would address their gaps/needs. In addition, the process was designed to encourage cross-departmental collaboration yet remain feasible to implement for regional staff. This helped promote uptake, as stakeholders recognized the value and potential from the beginning.
2. **Manage change and encourage new practices through frequent follow-up support:** Project staff were seconded to RHB/ZHD offices (as part of the overall project approach) and thus had regular contact with RHB/ZHD staff following orientation. They regularly provided mentorship on the overall process and helped troubleshoot technical issues during the roll-out, especially at the beginning.
3. **Embed implementation research into introduction processes:** When introducing tools into a system, embedding implementation research during roll-out is critical, as it can provide valuable information on what's working, what isn't, and how to adapt or improve.
4. **Don't let perfect be the enemy of the good:** As regular reporting had lapsed just before roll-out, negatively affecting the availability of data (particularly in BG), triangulation indicators could not be fully leveraged. Despite this limitation, we encouraged the group to start analyzing the data they had available, while simultaneously working to improve overall data availability. Getting started with imperfect data allowed stakeholders to begin to work collaboratively, increase their comfort and capacity to analyze triangulated data, and take action to address problems at the district level.

## WAY FORWARD AND RECOMMENDATIONS

Despite limitations in data availability, the process documentation showed some evidence that the use of the IDTT and data review process improved decision making and spurred actions that improved the management of immunization services at the district and health facility levels. Respondents also noted the value of regularly using triangulated data. KII respondents provided the following recommendations on the data triangulation tool and process.

- National-level MOH officials should institutionalize the tool for use at regional and zonal management levels.
- Formalize clear tasks/responsibilities of every data triangulation committee member from across departments.
- Improve the availability of vaccine supply data; more human and financial resources are needed to support this process.

The IDTT and review meeting process described in this article represents an approach to introducing a regular data triangulation process for immunization programs. These early findings and lessons related to introducing the tools and establishing regular processes show promise in the ability of EPIs to successfully use triangulated data to address challenges. The IDTT process facilitated collaboration and decision making, as well as breaking down of silos; more data use and attention to data availability and quality; and some demonstrable actions that improved the management of immunization services. More work needs to be done to document this kind of work, including at more advanced stages of implementation occurring over a longer period. If data triangulation processes advance and scale, they should be integrated into current health information systems, such as DHIS2, to be properly institutionalized and sustained.

## Supplementary Material

GHSP-D-21-00719-supplement-2.docx

GHSP-D-21-00719-supplement-1.xlsm

GHSP-D-21-00719-supplement-3.pdf

## Notes

**Peer Reviewed**

**First published online:** June 7, 2022.

## References

[1] UNICEF, World Health Organization (WHO). Progress towards global immunization Goals – 2019. Summary presentation of key indicators. Updated July 2020. Accessed May 17, 2022. https://cdn.who.int/media/docs/default-source/immunization/global_monitoring/slidesglobalimmunization.pdf

[2] Phase V (2021–2025). Gavi, the Vaccine Alliance. Updated June 9, 2021. Accessed May 17, 2022. https://www.gavi.org/our-alliance/strategy/phase-5-2021-2025

[3] World Health Organization (WHO). Immunization Agenda 2030: A Global Strategy to Leave No One Behind. WHO; 2020. Accessed May 17, 2022. https://www.who.int/teams/immunization-vaccines-and-biologicals/strategies/ia2030

[4] Ethiopia Ministry of Health (MOH). Health Sector Transformation Plan II (HSTP II): 2020/21-2024/25 (2013 EFY - 2017 EFY). MOH; 2021. Accessed May 17, 2022. https://www.familyplanning2020.org/sites/default/files/HSTP-II.pdf

[5] Triangulation for improved decision-making in immunization programmes. TechNet-21. Updated July 2020. Accessed May 17, 2022. https://technet-21.org/en/topics/triangulation

[6] PATH, Pan American Health Organization (PAHO). Immunization Data: Evidence for Action. A Realist Review of What Works to Improve Data Use for Immunization, Evidence from Low- and Middle-Income Countries. PATH, PAHO; 2019. Accessed May 17, 2022. https://www.path.org/resources/immunization-data-evidence-for-action-a-realist-review-of-what-works-to-improve-data-use-for-immunization-pr%C3%A9cis/

[7] Combining health and supply chain management in Lao with DHIS2. DHIS2. Accessed May 17, 2022. https://dhis2.org/lao-supply-chain/

[8] Kebede M Adeba E Chego M. Evaluation of quality and use of health management information system in primary health care units of east Wollega zone, Oromia regional state, Ethiopia. BMC Med Inform Decis Mak. 2020;20(1):107. 10.1186/s12911-020-01148-4. 32532256 PMC7291546

[9] Ethiopian Public Health Institute (EPHI), Ministry of Health (MOH), World Health Organization (WHO). Health Data Quality Review: System Assessment and Data Verification for Selected Indicators. EPHI, MOH, WHO; 2016. 10.13140/RG.2.2.21284.09604

[10] Ethiopia: WHO and UNICEF estimates of immunization coverage: 2019 revision. World Health Organization. Updated July 6, 2021. Accessed May 17, 2022. https://cdn.who.int/media/docs/default-source/country-profiles/immunization/immunization_eth_2021.pdf

[11] World Health Organization (WHO) Regional Office for Africa. Reaching Every District (RED): A Guide to Increasing Coverage and Equity in All Communities in the African Region. 2017 ed. WHO; 2017. https://www.afro.who.int/sites/default/files/2018-02/Feb%202018_Reaching%20Every%20District%20%28RED%29%20English%20F%20web%20v3.pdf

