# Supplementary material for: Early Lessons From Ethiopia in Establishing a Data Triangulation Process to Analyze Immunization Program and Supply Data for Decision Making
Source: Glob Health Sci Pract. 2022 Jun 29;10(3):e2100719. doi: 10.9745/GHSP-D-21-00719 (PMC9242614; doi:10.9745/GHSP-D-21-00719)
Supplement: GHSP-D-21-00719-supplement-2.docx [file GHSP-D-21-00719-supplement-2.docx]

Supplement 2. District level triangulation indicators

| Indicator | Data Sources | What it means |
| --- | --- | --- |
| Ratio of PCV1 doses administered to Penta1 doses administered | DHIS2 | PCV1 and Penta1 should be administered at the same time to a client; therefore, the number of doses administered of each antigen during the reporting period should be similar and the ratio close to 1. |
| Ratio of total Penta administered (doses 1, 2, and 3) to Penta issued  *Rolling average over six months | DHIS2 for Penta administered  mBrana for Penta issued | This ratio should be close to 1. It’s best to look at this over time, as stock can balance out, and to allow for buffer; therefore, this is calculated as a rolling average of the previous six months of data. (Note: Penta is in single dose vials.) |
| Ratio of total measles doses opened to total doses issued  *Rolling average over six months | DHIS2 for doses opened  mBrana for doses issued | Doses opened includes any wastage in addition to doses administered (measles vaccine comes in 10-dose vials). This ratio should be close to 1, but it’s best to look at it over time to allow for buffer; therefore, this is calculated as a rolling average of the previous six months of data. |
| Ratio of ending stock balance for Penta to buffer stock  *Compares end stock balance to buffer stock required | mBrana | This compares the ending stock balance at the district to the buffer stock required. |
| Doses administered for measles  *Rolling average over three months | DHIS2 | This takes a three-month rolling average of measles doses administered to assess if the number is relatively stable from month to month, or if there are large upward/downward variations. |
| Doses administered for IPV  *Rolling average over three months | DHIS2 | Similar to the indicator above, this takes a three-month rolling average of IPV doses administered to assess if the number is relatively stable from month to month, or if there are large upward/downward variations. It serves as another tracer vaccine. |
