# Supplementary material for: Early Lessons From Ethiopia in Establishing a Data Triangulation Process to Analyze Immunization Program and Supply Data for Decision Making
Source: Glob Health Sci Pract. 2022 Jun 29;10(3):e2100719. doi: 10.9745/GHSP-D-21-00719 (PMC9242614; doi:10.9745/GHSP-D-21-00719)
Supplement: GHSP-D-21-00719-supplement-3.pdf [file GHSP-D-21-00719-supplement-3.pdf]

Supplement to: Almiñana A, Bayeh A, Girma D, et al. Early lessons from Ethiopia in establishing a data triangulation process to analyze immunization program and supply data for decision making. Glob Health Sci Pract. 2022;10(3):e2100719. https://doi.org/10.9745/GHSP-D-21-00719

Immunization Data Triangulation Tool (IDTT)

User Guide

August 2020

Table of Contents

Acronyms 3
Introduction 4
Purpose of the guide 4
Audience for the guide 4
Part I: Why triangulate data 5
The role of data and benefits of triangulating 5
Types of decisions 6
Immunization data triangulation tool 7
Part II: Immunization data triangulation tool (IDTT): step by step instructions 7
General structure 7
Navigation 8
Downloading reports from DHIS2 9
Downloading reports from mBrana 14
DHIS2 data entry 16
mBrana data entry 17
Part III: Understanding the results 18
Regional dashboard: DHIS2/mBrana triangulation 18
Regional dashboard: RED categorization 19
Woreda dashboard 20
Woreda DHIS2/mBrana data triangulation table 20
Vaccine coverage table 22
Part IV: Data review and decision-making 22
IDTT data review team 22
IDTT data review meeting preparation and process 23
From analysis to action 24
Annex 1: Definitions of DHIS2 and mBrana triangulated indicators 25
Annex 2: Suggested data review meeting agenda 26
Annex 3: Suggested template for data review meeting notes 27

Acronyms

DOR	Drop out rate
EPI	Expanded Programme on Immunization
HSTP	Health Sector Transformation Plan
IDTT	Immunization Data Triangulation Tool
JSI	John Snow, Inc.
PSA	Pharmaceutical Supply Agency
RED	Reaching Every District
RHB	Regional Health Bureau
UI-FHS	Universal Immunization through Improving Family Health Services

Introduction

Strengthening data use and quality is critical to achieving high, equitable immunization coverage. Regional staff in Ethiopia have access to a lot of data on immunization, notably through DHIS2, which collects service statistics for all health facilities, and mBrana, an open-source mobile logistics management information system. The Immunization Data Triangulation Tool (IDTT) is an Excel tool that triangulates immunization program and supply chain data for improved decision-making and action. The tool compares key indicators from both data sources, provides scores based on performance, and provides decision support features such as recommended actions based on the scores. This user guide accompanies the IDTT as a helpful reference and resource.

This guide is divided into four major parts:

**Part I**	**Why triangulate data**<br>Describes the role of data in immunization and supply chain programs and the benefits of triangulating data
**Part II**	**Immunization data triangulation tool (IDTT): step by step instructions**<br>Provides overview of the structure of the tool and how to use it
**Part III**	**Understanding the results**<br>Describes interpretation of IDTT results
**Part IV**	**Data review and decision-making**<br>Outlines the data review meeting process

Purpose of the guide

The purpose of this guide is to:

- Provide information on the rationale and process for conducting data triangulation across immunization and supply chain indicators
- Instruct users on how to use the Excel-based Immunization Data Triangulation Tool (IDTT) developed by JSI/UI-FHS, in order to comprehensively assess supply chain and program performance in each woreda in the region
- Guide the data review process to facilitate decision-making based on triangulated data

These efforts aim to empower regional level staff to strengthen data quality, analysis, and use for improved immunization service delivery and supply chain management.

Audience for the guide

This guide is intended to be used by regional level staff engaged in the Expanded Programme on Immunization (EPI), Supply Chain (PSA), and Planning & Resource (DHIS2) departments who will use the tool or take part in data review meetings.

Part I: Why triangulate data

The role of data and benefits of triangulating

As immunization programs strive to reach equitable, high coverage in all districts, the role of strengthening data quality and use is increasingly important. Ethiopia's Health Sector Transformation Plan (HSTP) has also recognized the pivotal role of data through its "information revolution" transformation agenda, noting: "all functions of the health system rely on the availability of timely, accurate and dependable information for decision-making. Hence, revolutionizing the existing practice of collecting, analysing, disseminating and utilising information in the health sector can considerably contribute towards holistic transformation."[1]

Using data in a systematic way can improve decision-making, but it is not always easy to do. Regional staff in Ethiopia have access to a lot of data on the immunization program, most notably through DHIS2, which collects service statistics for health facilities across the entire country. However, data in DHIS2 are limited by several factors, including use of uncertain denominators, unintended incentives for over reporting, and poor data management practices. The quality of immunization data and the limitations of any individual dataset therefore require the review of multiple data sources, in order to interpret and use program data more effectively.

Data triangulation is the synthesis of data from two or more existing data sources to address relevant questions for program planning and decision making.[2]

Data triangulation uses multiple data sources to get better reliability/accuracy. It adds a layer of validation, counterbalancing one inaccurate or incomplete data set by validating it against others. This can provide an additional layer of insight and understanding, turning various pieces of data into information that can be used to make better decisions.

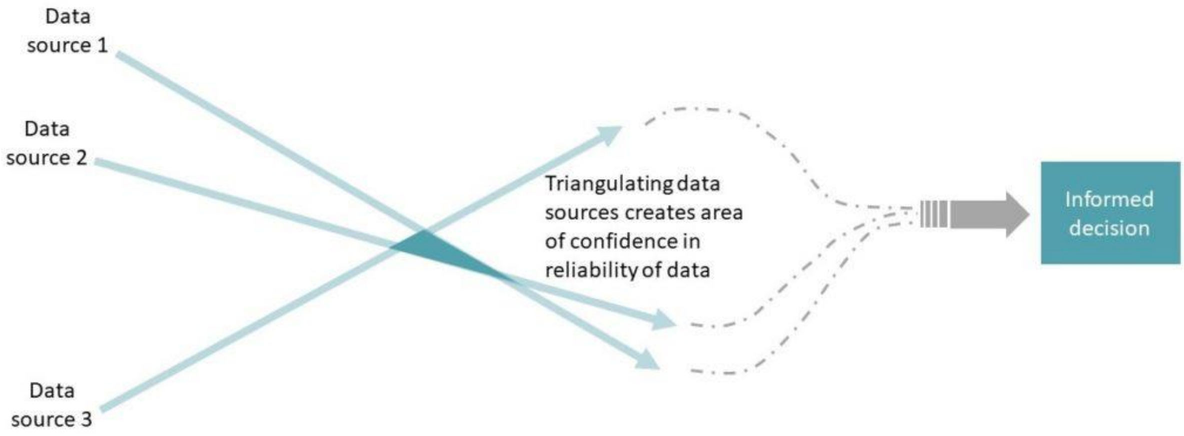

Image adapted from: INTRAC for civil society

[1] Federal Ministry of Health, Ethiopia. Health Sector Transformation Plan 2008-2012 EFY. Available online.
[2] WHO, UNICEF, and CDC. General Triangulation Guidance: Sub-national Level. Draft May 2020. Available online.

For example, triangulating administrative immunization data and vaccine stock data provides better insight into program performance than looking at either data source by itself because it helps complete the whole picture; a drop in the number of doses being given could be explained by a stock out. These two sets of data also make sense to triangulate against each other because they have similar reporting periods/frequency, or may use similar denominators for planning purposes.

Data alone cannot improve immunization coverage. But it can strengthen understanding of the immunization program (particularly in places where data quality is weak), improve planning and coordination, and provide information that can enhance performance at lower levels.

Thus, data triangulation is an approach to gain more insight and drive decisions based on data that will improve programs.

Types of decisions

There are different types of decisions that can be made to try and improve a program:

- Operational Decisions: day-to-day decisions that address immediate issues or needs.
- Performance Improvement Decisions: Decisions that address the root cause by changing a process or optimizing the supply chain or program activities. May be longer-term, take more analysis, and may involve more people.

Here are some examples:

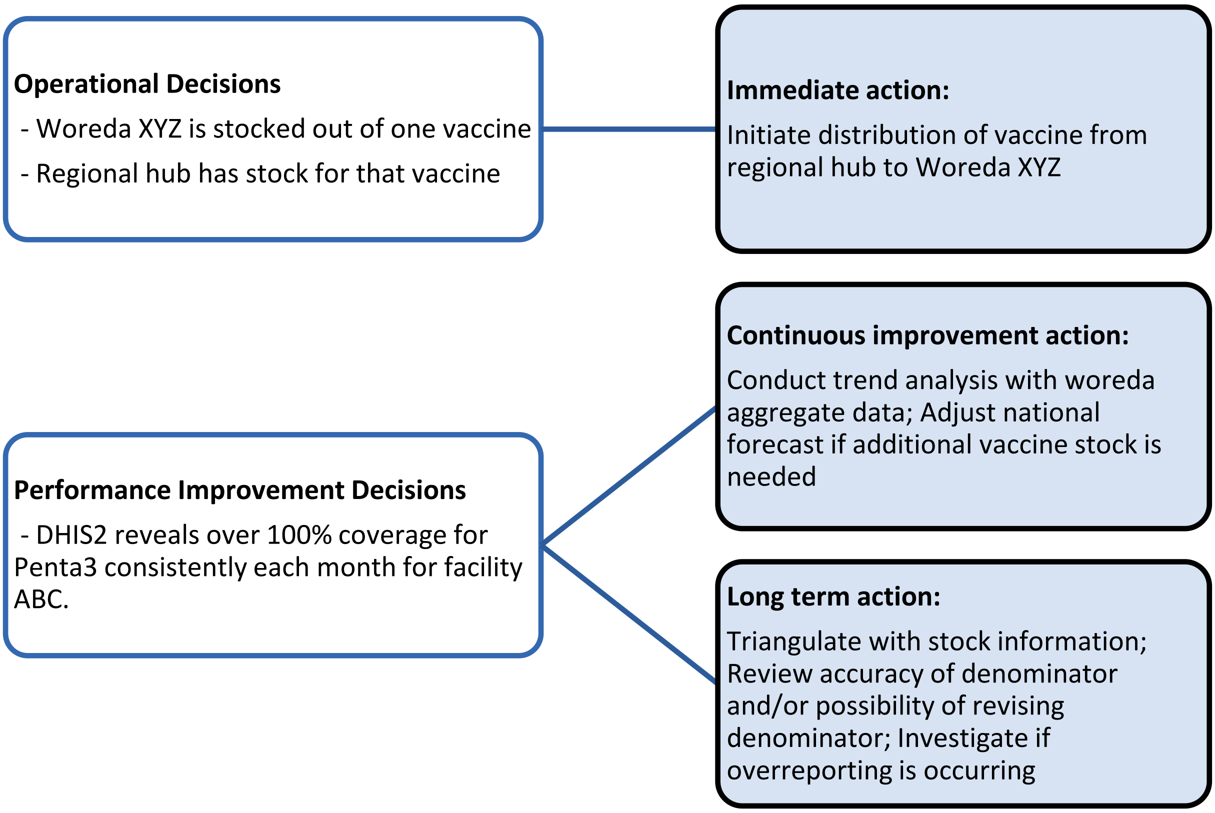

Through continued regular review and group discussion of the data triangulated with the IDTT, regional level staff will be able to influence and take action on both of these types of decisions to address immediate and longer-term issues.

Immunization data triangulation tool

The IDTT is a joint effort by JSI projects focused on facilitating the use of data to improve immunization and the supply chain in Ethiopia.

JSI's UI-FHS project has used the innovative "RED-QI" approach to improve equitable access to routine immunization (RI) services for all eligible children in Ethiopia, including those in hard-to-reach pastoralist communities. The approach helps health personnel plan, implement, and monitor tailored health services to reach all children with RI, regardless of where they live. The project currently supports various efforts to build capacity within Regional Health Bureaus for improved management of immunization services, including in the use of data for decision-making.

JSI's Vaccine Supply Chain project has developed mBrana, an open-source mobile logistics management information system. mBrana is currently being deployed to woredas to improve vaccine commodity management and increase data visibility between central, regional ("hub"), and woreda level. However, use of vaccine supply chain data by program staff (i.e EPI focal points) has been limited to date, and is recognized as a gap in the immunization program.

The IDTT links DHIS2 and mBrana data and triangulates data against each other.

It aims to triangulate data across programs and data sources in a way that is simple, feasible, and drives action that regional level staff can be responsible for.

The IDTT was developed drawing from direct experiences with JSI staff and RHB personnel, including a consultative workshop with experts from the Benishangul Gumuz Regional Health Bureau.

Part II: Immunization data triangulation tool (IDTT): step by step instructions

General structure

The IDTT is a Microsoft Excel workbook that consists of the following worksheets (tabs):

Worksheet name	Description
**Cover page**	Provides the user with basic information on the tool and contains navigation buttons. The user selects the region, month, and year to analyze and starts on this page.

Worksheet name	Description
**DHIS2 data entry**	User copies the relevant data from the DHIS2 monthly report, pastes it into the indicated section, and submit it on this page.
**mBrana data entry**	User copies the relevant issues data and stock on hand data from mBrana, pastes it into the indicated sections, and submit it on this page.
**Regional Dashboard: DHIS/mBrana**	Provides a summary of performance by zone for all woredas in the region, and it provides a data triangulation composite score (made up of 6 key indicators) for each woreda.<br><br>This dashboard is automatically populated once data entry is complete.
**Regional Dashboard: RED Categorization**	Provides a summary by zone of immunization coverage analysis using RED Categorization and provides the RED Categorization score for each woreda.<br><br>This dashboard is automatically populated once data entry is complete.
**Woreda Dashboard**	Provides detailed data for a woreda for key indicators related to immunization/supply chain data triangulation, coverage, and dropout.
**Indicator definitions**	Provides detailed calculations and definitions for displayed indicators.
**Credits**	Displays credits/acknowledgements for the tool and contact information.

Navigation

Users can navigate from one tab to another as you would in any other Excel workbook by selecting a tab at the bottom of the workbook, and also by utilizing the buttons on the screen (see examples in Figure 1.

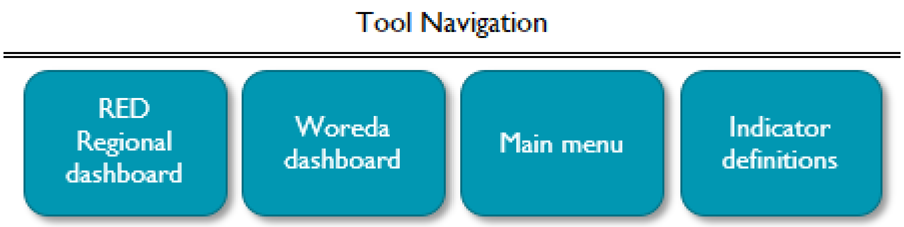

It is recommended that you advance through the worksheets in the order they appear in the workbook.

Supplement to: Almiñana A, Bayeh A, Girma D, et al. Early lessons from Ethiopia in establishing a data triangulation process to analyze immunization program and supply data for decision making. Glob Health Sci Pract. 2022;10(3):e2100719. https://doi.org/10.9745/GHSP-D-21-00719

Downloading reports from DHIS2

In order to complete data entry in the IDTT, you must first download the relevant reports from DHIS2. These downloaded reports will be subsequently be copy-pasted into the data entry page of the IDTT.

To download the report correctly from DHIS2, follow these instructions:

Step 1:
Navigate to the website https://dhis.moh.gov.et/ and login using your given user name and password

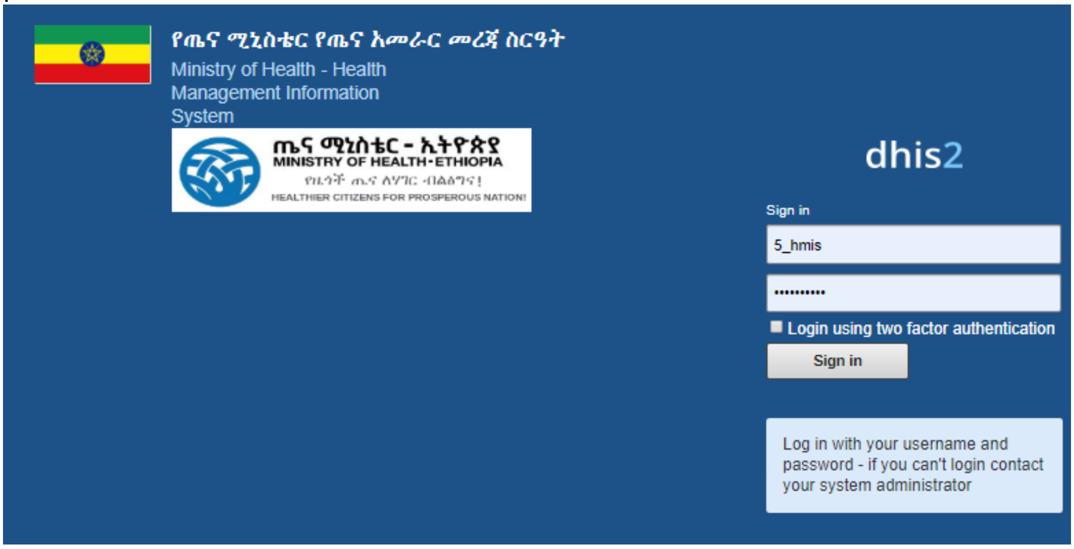

Step 2:
Click on "apps" in the top-right corner and choose "pivot table"

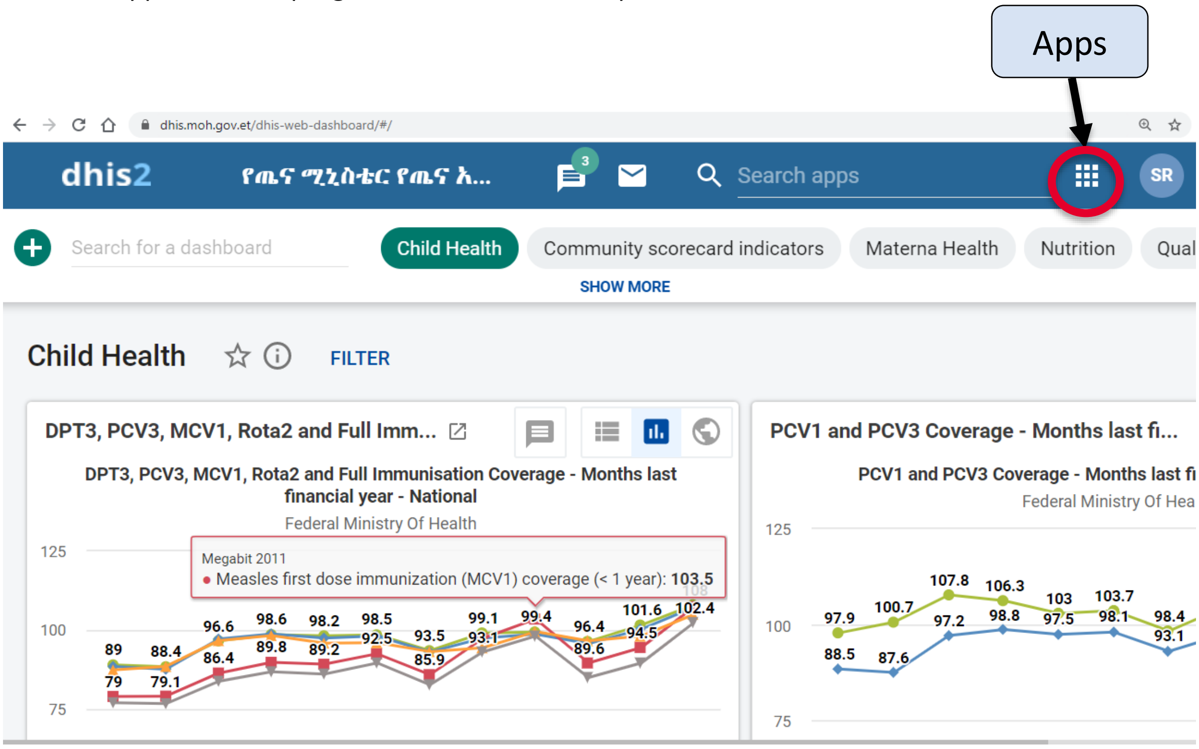

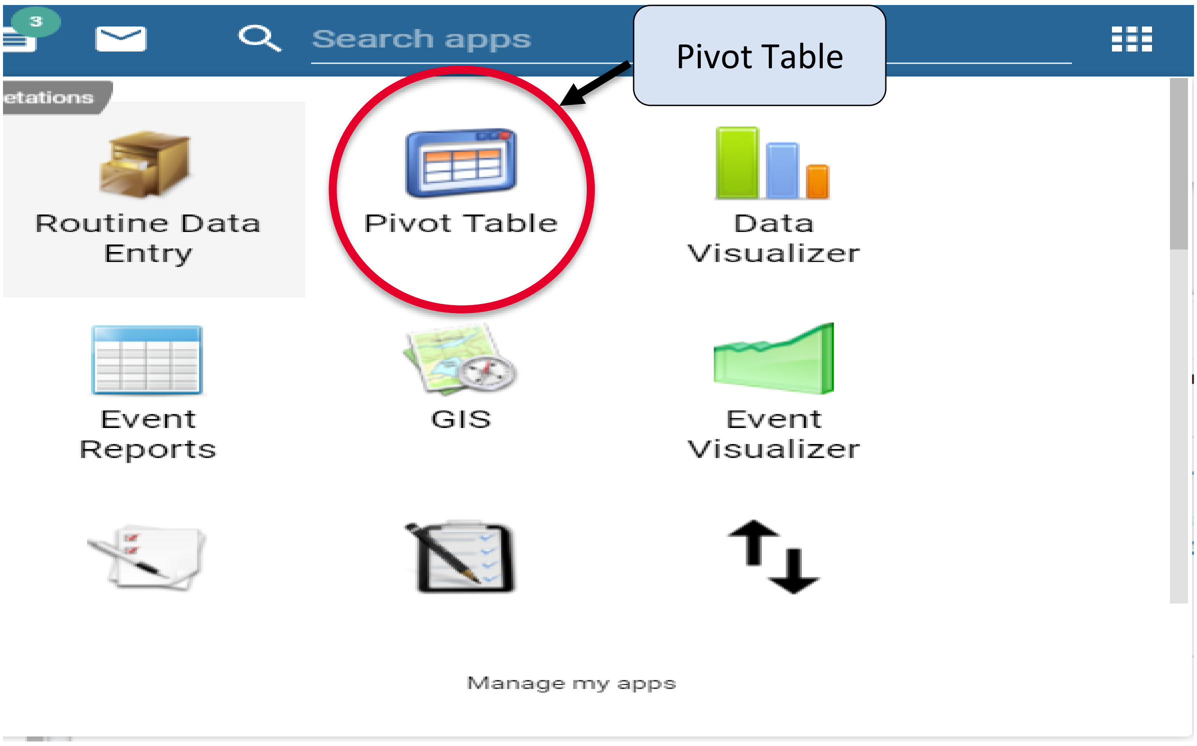

Step 3:
Select the required three dimensions:

Data captured in DHIS 2 needs at least three dimensions that answer three questions: the **WHAT**, the **WHEN** and the **WHERE**

**WHAT**	Capture what data is recorded in the system.<br><br>E.g. number of BCG doses given, number of ANC4 visits
**WHEN**	The period the activity is performed. There are a lot of options to choose a time period like monthly, quarterly, yearly, last 3 months.<br><br>E.g. 2012, June 2012, Last 6 months
**WHERE**	The organization/ facility that performed the activity.<br><br>E.g. Somali regional HB, Bullen WorHO, Afambo Health center

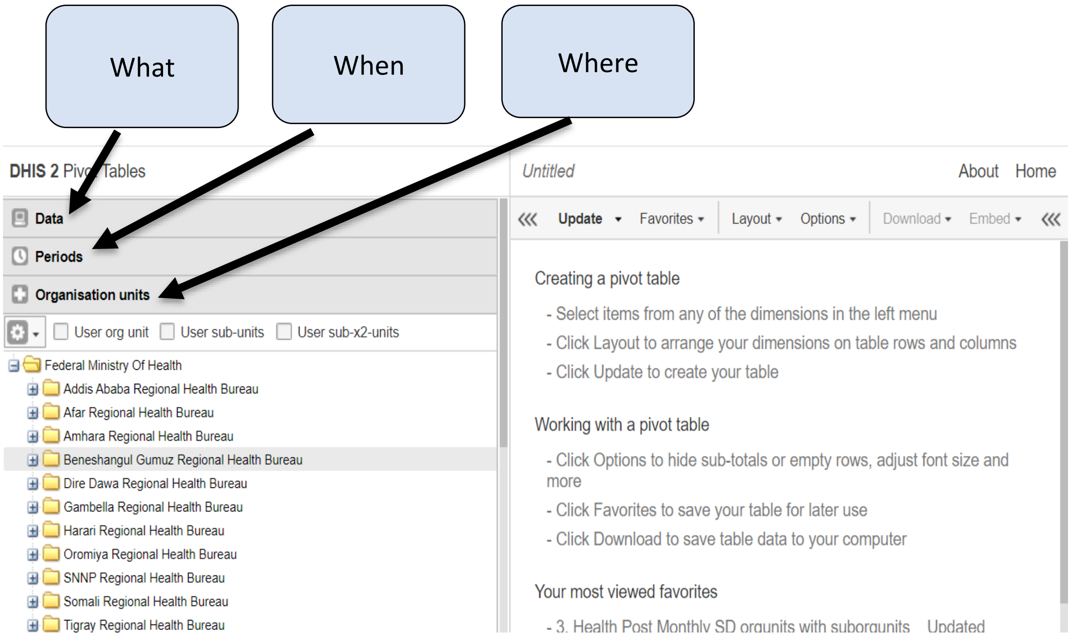

<u>What</u>

First choose data elements from ***Data*** and select data element group from **Data element lists.** Note that RI related indicators are found under 02 – Neonatal and child health, finally select the required 14 data elements from 02 – Neonatal and child health. We can select these indicators using two methods:

1. Using searching method. Write the name of the indicator on the search area
2. Double click on the required indicators from the list and make sure that this indicator is included on the selected box as shown below.

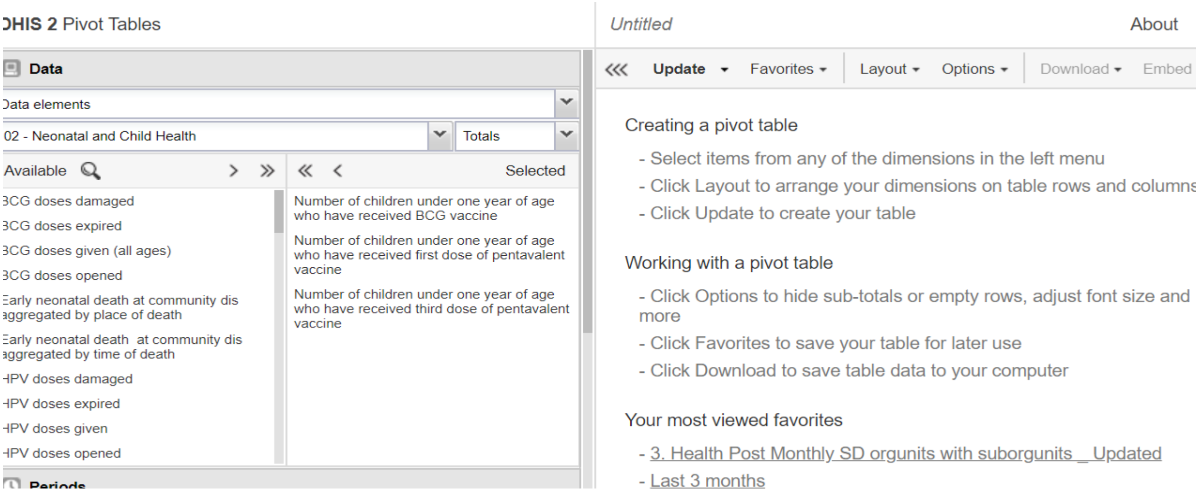

Note: Here you need to select the following 17 indicators for the data triangulation purpose

1. Number of children under one year of age who have received BCG vaccine
2. Number of children under one year of age who have received first dose of pentavalent vaccine
3. Number of children under one year of age who have received third dose of pentavalent vaccine

4. Number of children under one year of age who have received first dose of pneumococcal vaccine
5. Number of children under one year of age who have received third dose of pneumococcal vaccine
6. Number of children under one year of age who have received first dose of rotavirus vaccine
7. Number of children under one year of age who have received second dose of rotavirus vaccine
8. Number of children under one year of age who have received first dose of polio vaccine
9. Number of children under one year of age who have received third dose of polio vaccine
10. Number of children under one year of age who have received one dose of inactivated polio vaccine
11. Number of children under one year of age who have received first dose of measles vaccine
12. Number of children under two years of age who have received second dose of measles vaccine
13. Number of children received all vaccine doses before 1st birthday
14. Number of Infants whose mothers had protective doses of TT against NNT (PAB)
15. Pentavalent (DPT-HepB-Hib) doses given (all ages)
16. Measles doses opened
17. Pentavalent (DPT-HepB-Hib) doses opened

<u>When</u>
There are different types of options for selecting time period; note that last 12 months is selected by default and needs to be un-ticked it if we don't need that period of time.

Note: Here we should select Monthly as data triangulation is conducted on monthly basis.

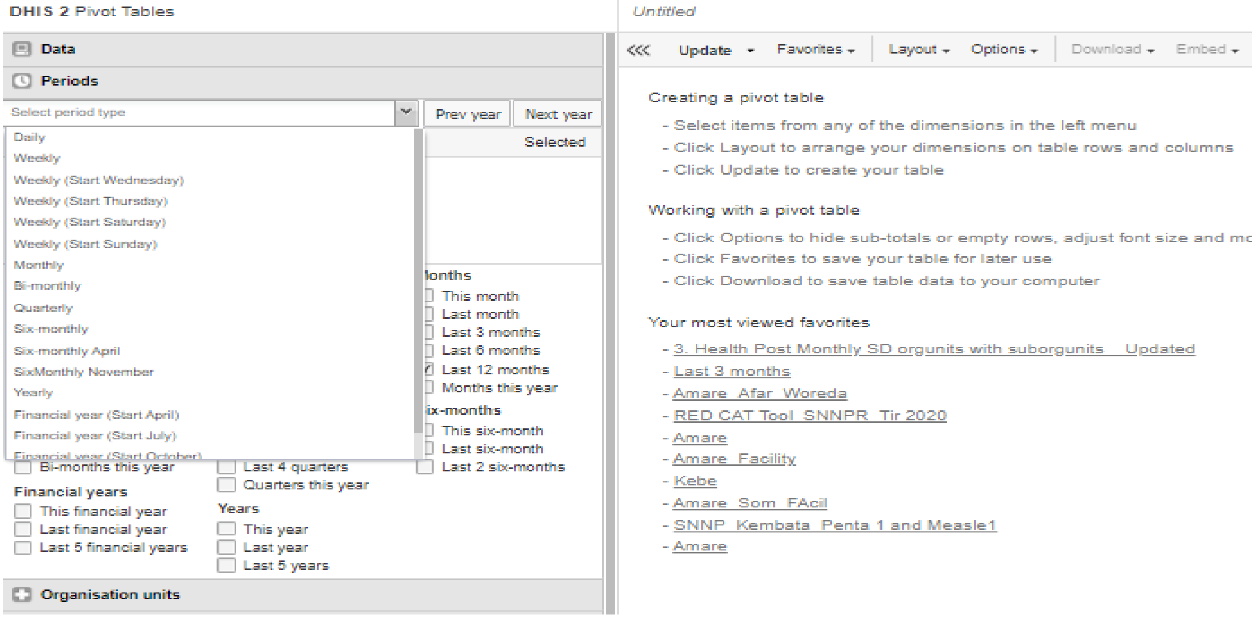

<u>Where</u>
Select Organization unit.
Note: Select WoHO under organization units to have regional data by woreda

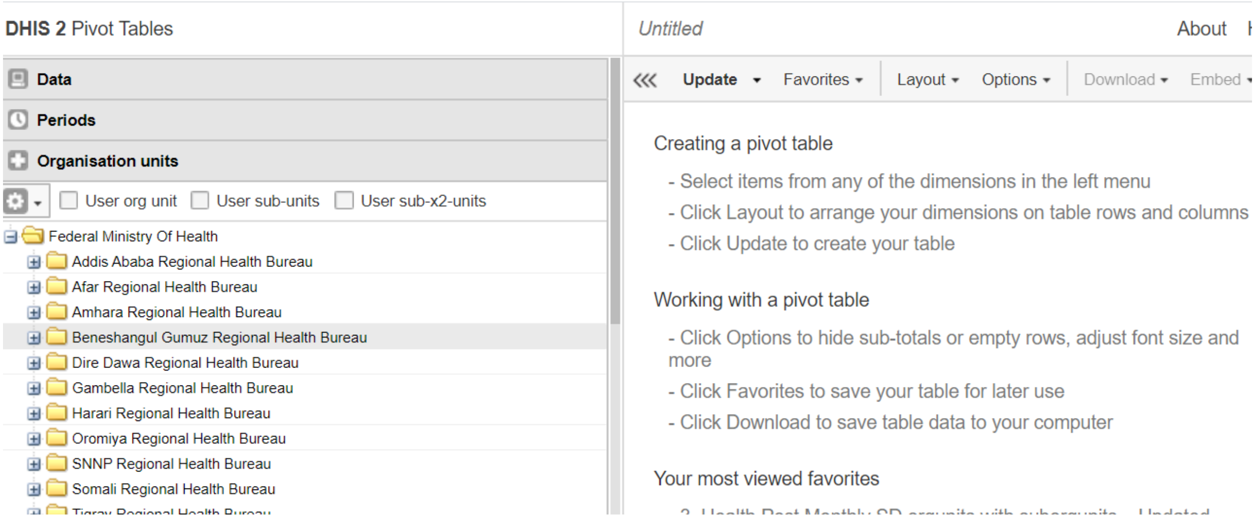

Step 4:

Click Update.

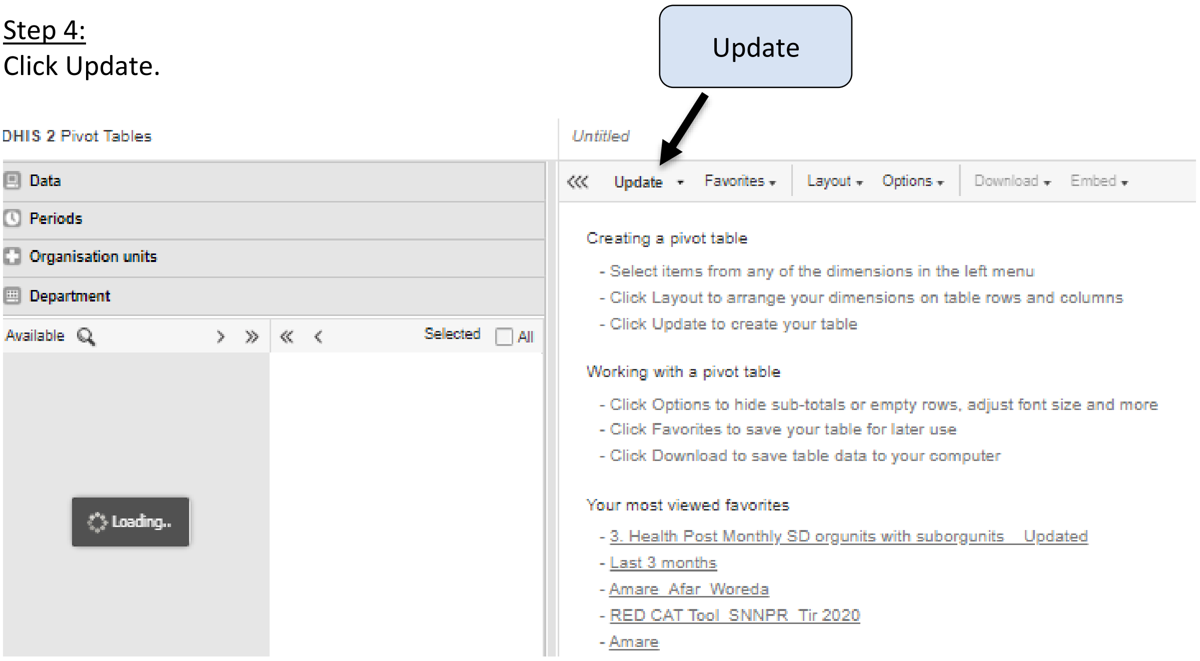

Step 5:

Click on download, choose Excel to download the file using Excel, and save it at the place where you want to document the file.

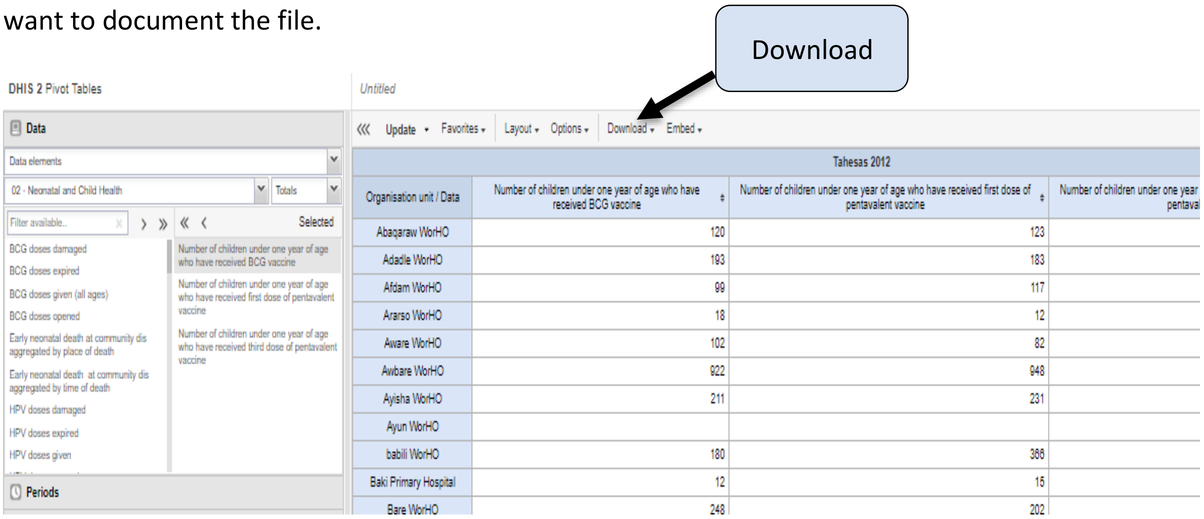

Supplement to: Almiñana A, Bayeh A, Girma D, et al. Early lessons from Ethiopia in establishing a data triangulation process to analyze immunization program and supply data for decision making. Glob Health Sci Pract. 2022;10(3):e2100719. https://doi.org/10.9745/GHSP-D-21-00719

This is what the downloaded excel file looks like (example from Benishangul Gumuz region):

A1 Yekatit 2012

	A	B	C	D	E	F	G	H	I	J	K	L	M	N	O	P	Q	R
1	Yekatit 2012																	
2																		
3	organisatic	Number of	Number of	Number of	Number of	Number of	Number of	Number of	Number of	Number of	Number of	Number of	Number of	Number of	TT doses g	Pentavaler	Measles d	Pentavalent (D
4	Agalo Meti	51	79	80	79	80	79	64	79	80	80	66	32	66	182	167	140	167
5	Asosa Tow	213	200	195	200	195	200	189	200	195	195	193	154	193	500	584	390	584
6	Assosa W	169	188	195	188	195	188	187	188	195	195	202	161	202	639	570	540	570
7	Bambasi V	142	164	147	164	147	164	129	164	147	147	117	65	117	112	440	260	443
8	Belo Jegar	65	99	77	99	77	104	85	99	77	77	66	42	82	225	261	140	261
9	Bulen Wor	210	142	176	142	176	142	165	142	176	173	143	126	135	267	491	360	495
10	Dangur Wc	97	137	137	137	137	126	125	137	137	140	78	39	73	236	389	169	402
11	Dibate Wo	245	204	225	204	225	204	239	204	225	207	250	213	213	393	674	650	687
12	Guba Worl	70	71	79	71	82	72	72	72	80	61	46	6	50	134	186	91	178
13	Homosha '	86	62	66	62	66	64	57	62	66	57	56	66	56	210	179	170	179
14	Kamashi F																	
15	Kamashi V	47	61	59	61	59	61	47	61	59	59	27	22	19	176	132	90	132
16	Kurmuk W	20	42	48	42	48	42	59	42	48	48	42	43	51	123	149	90	149
17	Mandura V	77	102	94	102	94	102	83	102	94	94	81	50	50	101	257	143	257
18	Mao Komc	169	167	154	167	154	154	161	162	159	11	135	60	135	690	305	260	320
19	Menge Wc	241	209	152	209	152	209	214	209	152	152	191	147	191	441	575	410	575
20	Oda Bilidig	163	211	233	211	233	211	197	211	233	233	200	164	200	560	641	435	641
21	Pawi Hosp	25	51	38	51	38	51	51	51	38	39	42		42	128	140	50	140
22	Pawi Work	83	135	150	135	150	135	122	135	150	151	146	63	146	152	418	310	418
23	Sedal Wor	30	58	58	58	50	58	58	58	58	50	33		183	146	166	70	166
24	SherKole \	113	116	135	116	135	116	132	116	135	135	118	45	123	161	402	210	402
25	Wembera '	43	103	129	103	129	103	87	103	129	124	135	62	120	222	319	270	319
26	Yaso Work	34	113	58	113	58	109	81	109	58	75	47		61	84	213	66	213
27																		

You will copy and paste data from this downloaded Excel file onto the IDTT DHIS2 data entry worksheet (see instructions in below DHIS2 data entry section).

Downloading reports from mBrana

In order to complete data entry in the IDTT, you must first download the relevant reports from mBrana. These downloaded reports will be subsequently be copy-pasted into the data entry page of the IDTT.

To download the report correctly from mBrana, follow these instructions:

Step 1:
Login to mBrana Vaccine Dashboard website http://vaccine.mbrana.org/#!/login using your Username and password.

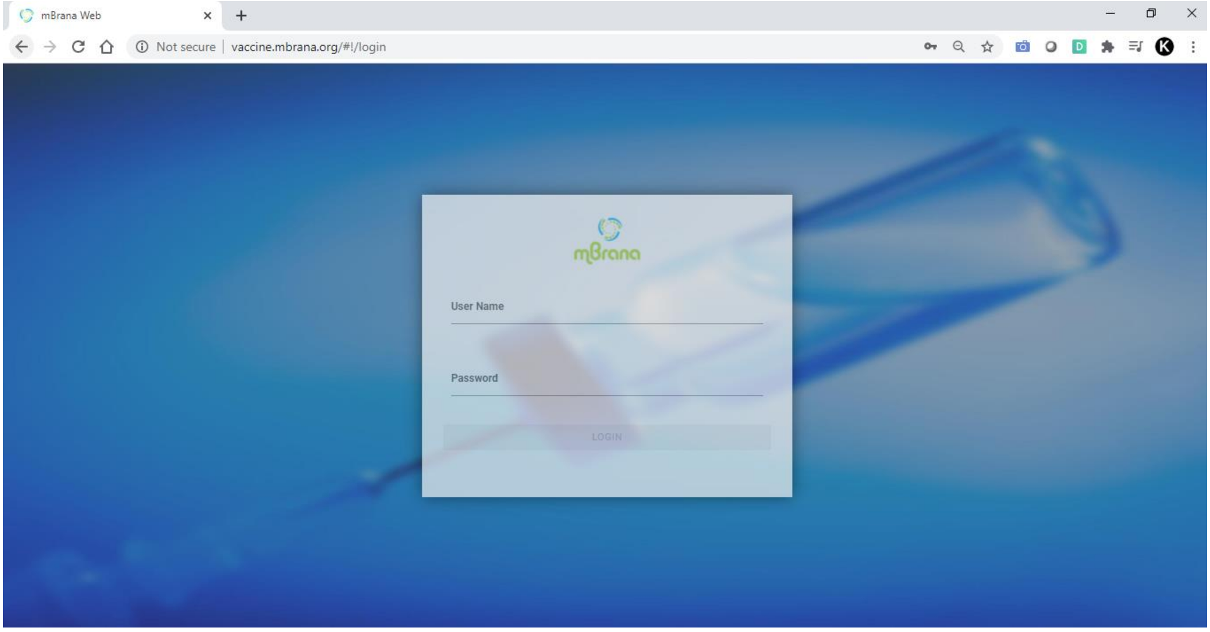

<u>Step 2 – SOH Report:</u>

In order to find an SOH in end of a month:

a) Go to SOH label in the left side of the dashboard
b) Then select a Region /Supplier Hub/Zone as needed
c) Finally enter the date you want to see the SOH

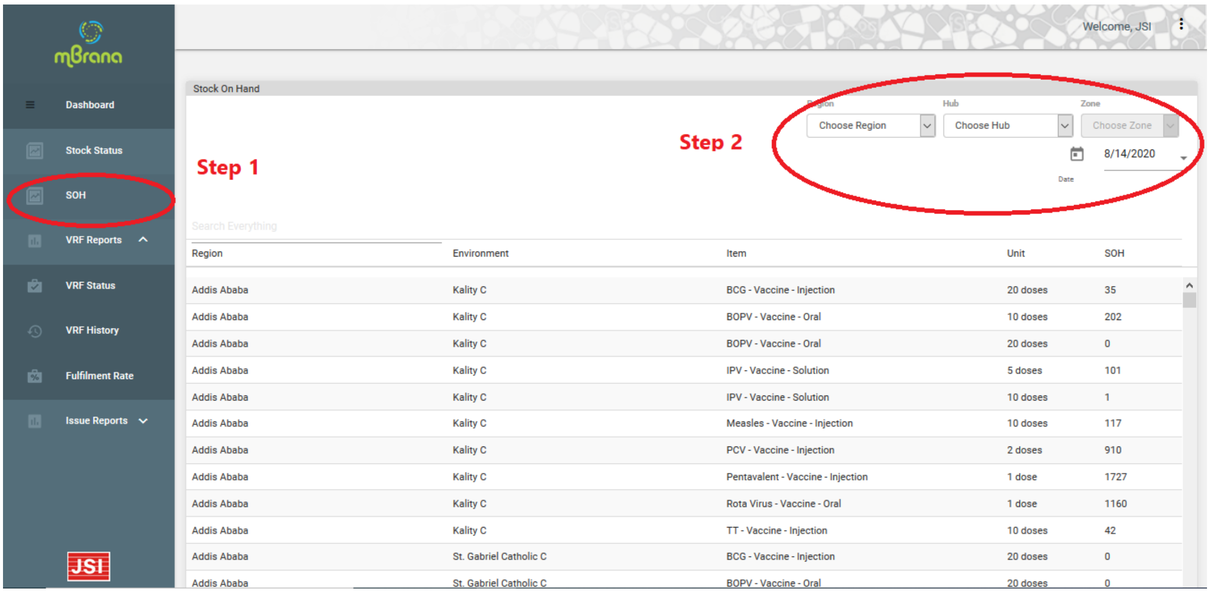

After you filter your results click Export to download as an Excel file. The file will automatically download to have data on pentavalent and measles vaccine.

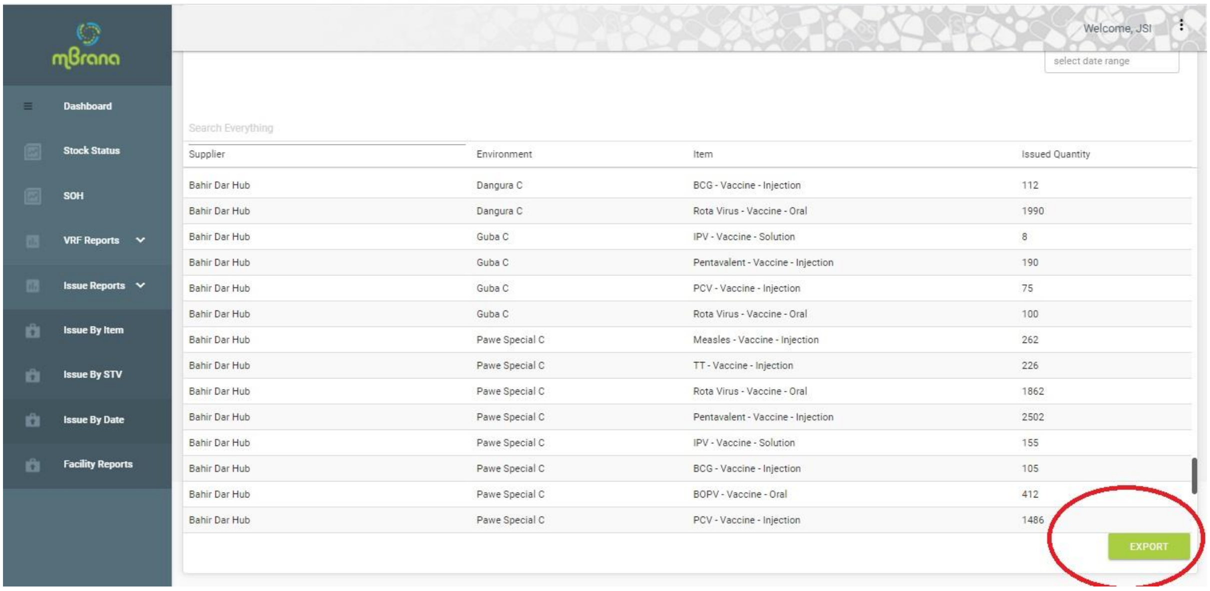

<u>Step 3 – Issues report:</u>

In order to find Issued Item within a specific time period:

a) Go to Issue Reports
b) Select Issue by Date
c) Then select a Region /Supplier Hub/Zone as needed
d) Select a specific time range and apply the dates.
e) Then select a Region /Supplier Hub/Zone as needed

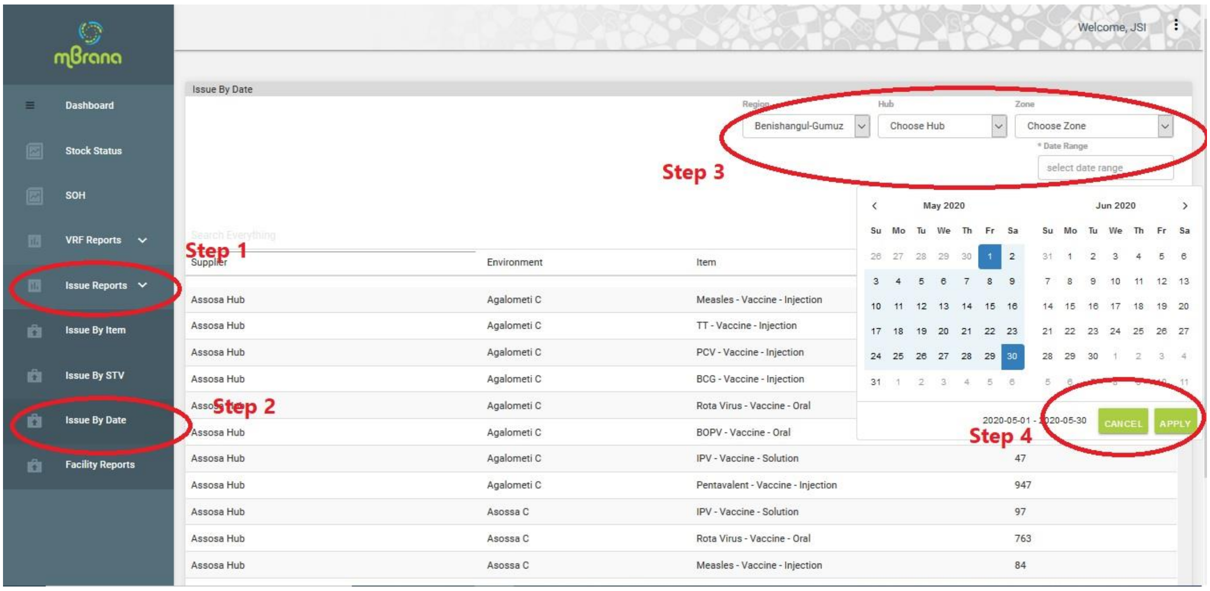

After you filter your results click Export to download as an Excel file. The file will automatically download to have data on pentavalent and measles vaccine.

DHIS2 data entry

Once the appropriate data has been downloaded, you will use the DHIS2 data entry worksheet to submit DHIS2 data.

Begin by selecting the month and year of the downloaded DHIS2 data you will be submitting.

Copy the relevant data from the DHIS2 monthly report; do not include the headers from the downloaded data – only include the data itself.

TIP: There is no need to copy the headers, only copy the data itself from the DHIS2 report.

To paste, right-click and choose the "paste as values" option.

After copying the data, select the highlighted cell in the data entry section and paste the data using the "paste as values" option. To paste as values, select the cell, right-click, and choose "paste as values" option under the paste options.

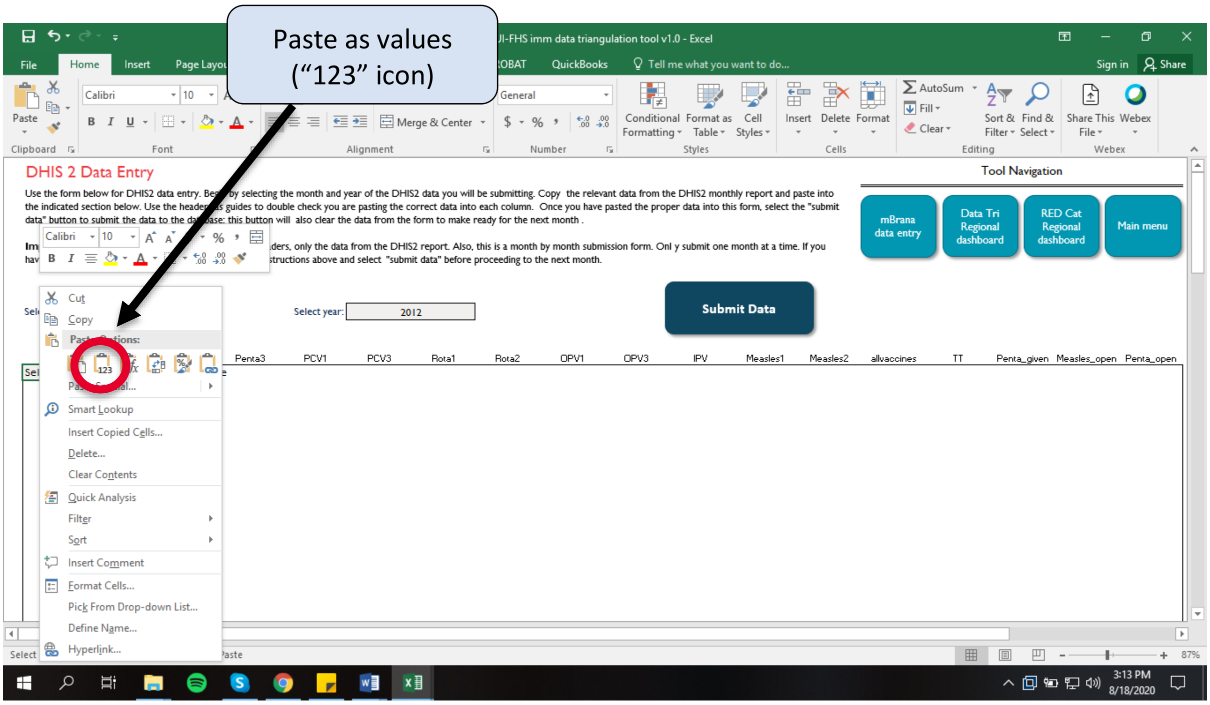

Use the headers as guides to double check you are pasting the correct data into this tool.

Once you have pasted the proper data into this form, select the "submit data" button to submit the data; this button will also clear the data from the form to make ready for the next month.

This is a month by month submission form. Only submit one month at a time. If you have a need to submit more than one month, follow the instructions above and select "submit data" before proceeding to the next month.

mBrana data entry

Once the appropriate data has been downloaded, you will use the mBrana data entry worksheet to submit the mBrana data.

Begin by selecting the month and year of the mBrana data you will be submitting.

Copy the relevant data (i.e., vaccines issues and ending month balance for Penta and Measles) from the mBrana report and paste into the indicated sections - **note that the forms for issues and end of month balance are <u>separate</u> entry points.**

Supplement to: Almiñana A, Bayeh A, Girma D, et al. Early lessons from Ethiopia in establishing a data triangulation process to analyze immunization program and supply data for decision making. Glob Health Sci Pract. 2022;10(3):e2100719. https://doi.org/10.9745/GHSP-D-21-00719

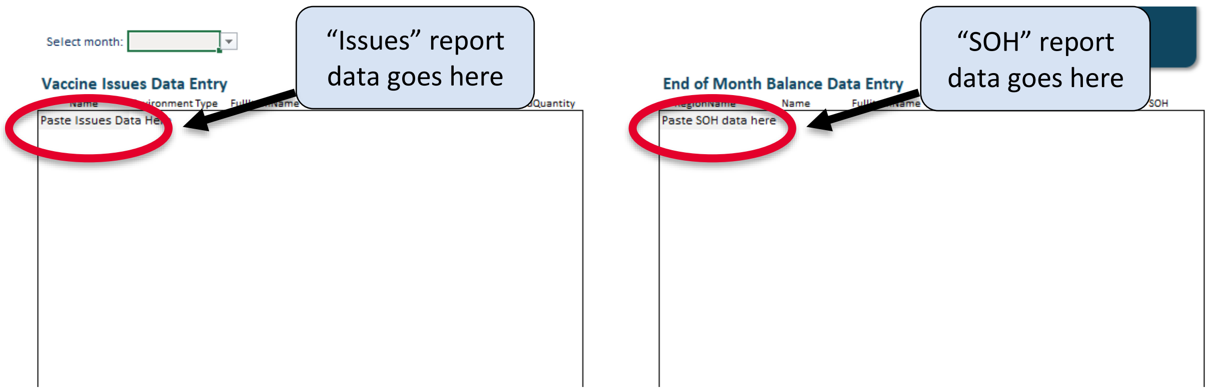

Use the headers as guides to double check you are pasting the correct data into this tool.

Once you have pasted the proper data into this form, select the "submit data" button to submit the data; this button will also clear the data from the form to make ready for the next month.

TIP: There is no need to copy the headers, only copy the data itself from the DHIS2 report.

To paste, right-click and choose the “paste as values” option.

This is a month by month submission form. Only submit one month at a time. If you have a need to submit more than one month, follow the instructions above and select "submit data" before proceeding to the next month.

Part III: Understanding the results

Regional dashboard: DHIS2/mBrana triangulation

The DHIS2/mBrana regional dashboard has two tables:

1) a summary by zone of the # of woredas with green, yellow, or red DHIS2/mBrana triangulation composite scores (for the most recent month), and
2) a main table with a composite score for every woreda for the previous six months.

The main table provides a snapshot of all woredas in this region using a green (strong), yellow (moderate), or red (poor) composite score. This composite score made up of six key indicators calculated for each woreda (see woreda dashboard section below for more details).

TIP: What does “missing data” mean?
When you see “missing data” displayed for a woreda, it means that data from 1 or more of the 6 indicators required to calculate the woreda’s composite score was missing. Check the woreda dashboard for that woreda to learn more: you can still review performance for the indicators that were not missing.

Based on the scores in the table, you can prioritize which woredas to analyze further through the woreda dashboard.

Regional dashboard: RED categorization

This dashboard displays RED Categorization analysis results. Similar to the other Regional Dashboard, this page displays two tables:

1) a summary by zone of the # of woredas with RED Category 1, 2, 3, or 4 (for the most recent month), and
2) a main table displaying the RED Categorization for every woreda, for the previous six months.

RED Categorization quickly analyzes and categorizes coverage and drop out rates (DOR) to assess whether a woreda has good/poor access of services and good/poor utilization of services.

There are four categories defined as follows[2]:

Category	Description
**1** (No problem)	Penta1 coverage is high (≥90%) – **good** access<br>Penta1-Penta3 DOR is low (<5%) – **good** utilization
**2** (Utilization problem)	Penta1 coverage is high (≥90%) – **good** access<br>Penta1-Penta3 DOR is high (≥5%) – **poor** utilization
**3** (Access problem)	Penta1 coverage is low (<90%) – **poor** access<br>Penta1-Penta3 DOR is low (<5%) – **good** utilization
**4** (Access & utilization problem)	Penta1 coverage is low (<90%) – **poor** access<br>Penta1-Penta3 DOR is high (≥5%) – **poor** utilization

Based on the results of the RED Categorization, there are various possible actions woredas may consider taking to help address EPI issues. Below are some possible actions for each category:

Category	Possible Actions
**1**<br><br>(good access, good utilization)	• Strengthen good practices<br>• Document and share best practices<br>• Validate data<br>• Recognize and reward good performers

[2] The WHO AFRO RED Guide (2017 version) denotes RED Categorization cutoff values of 80% Penta1 coverage and 10% DOR. However, many regions in Ethiopia are using 90% Penta1 coverage and 5% DOR as practical working cutoff values.

Supplement to: Almiñana A, Bayeh A, Girma D, et al. Early lessons from Ethiopia in establishing a data triangulation process to analyze immunization program and supply data for decision making. Glob Health Sci Pract. 2022;10(3):e2100719. https://doi.org/10.9745/GHSP-D-21-00719

Category	Possible Actions
**2**<br><br>(good access, poor utilization)	• Discuss with Quality Improvement Teams (QITs)/command posts to examine issues with the quality of service delivery<br>• Conduct supportive supervision/on the job aid, refresher trainings, review meetings focused on improving the quality of services<br>• Screen children at OPD to reduce missed opportunities (integration)<br>• Register and track drop outs<br>• Review regularity of outreaches, mobile activities<br>• Review planning for logistics to avoid stock outs<br>• Improve inter-personal communication and ensure caretakers know when to return for follow-up visits
**3**<br><br>(poor access, good utilization)	• Discuss with Quality Improvement Teams (QITs)/command posts on ways to improve access to immunization services<br>• Conduct supportive supervision/on the job aid, refresher trainings, review meetings focused on expanding reach of services<br>• Review static/outreach functionality (transport, staff, logistics, locations of outreaches, etc.)<br>• Re-map the catchment area to identify under-served communities<br>• Increase social mobilization<br>• Identify and track all pregnant women (register all infants to prevent left-outs)<br>• Give feedback to the local leaders
**4**<br><br>(poor access, poor utilization)	• Review EPI service delivery at all levels (review microplan):<br>○ Accessibility, static/outreach functionality (transport, staff, locations of outreaches, mobile, etc.)<br>○ Logistics, vaccines and gas, etc.<br>○ Data collection, analysis and utilization<br>• Discuss with Quality Improvement Teams (QITs)/Health Development Army/command posts/other community structures and community leaders to address both the reach and quality of immunization services<br>• Strengthen supportive supervision<br>• Identify and track pregnant women (register all infants to prevent left-outs)<br>• Improve inter-personal communication and ensure caretakers know when to return for follow-up visits<br>• Re-map the catchment area to identify under-served communities<br>• Register and track drop-outs

Woreda dashboard

Woreda DHIS2/mBrana data triangulation table

This table displays six key indicators for the woreda, with its performance for each over the previous six months. Four of the six indicators are ratios, which divide one indicator by another indicator that theoretically should be equal or similar.

Information about the six woreda-level indicators is included in the table below.

Indicator	Data sources	What it means
Ratio of PCV1 doses administered to Penta1 doses administered	DHIS2	PCV1 and Penta1 should be administered at the same time to a client; therefore, the number of doses administered of each antigen during the reporting period should be similar and ratio close to 1.
Ratio of total Penta administered (doses 1, 2, and 3) to Penta issued<br>**Rolling average over 6 months*	DHIS2 for Penta administered<br><br>mBrana for Penta issued	This ratio should be close to 1.0. It's best to look at this over time as stock can balance out and to allow for buffer; therefore, this is calculated as a rolling average of the previous 6 months' of data. (Note: Penta is in single dose vials)
Ratio of total measles doses opened to total doses issued<br>**Rolling average over 6 months*	DHIS2 for doses opened<br><br>mBrana for doses issued	Doses opened includes any wastage as well as doses administered (measles comes in 10 dose vials). This ratio should be close to 1.0, but it's best to look at it over time to allow for buffer; therefore, this is calculated as a rolling average of the previous 6 months' of data.
Ratio of ending stock balance for Penta to buffer stock<br>**Compares end stock balance to buffer stock required*	mBrana	This compares the ending stock balance at the woreda to the buffer stock required. The ratio should be larger than 1.
Doses administered for measles<br>**Rolling average over 3 months*	DHIS2	This takes a 3-month rolling average of measles doses administered to assess if the number is relatively stable from month to month, or if there are large upward/downward variations.
Doses administered for IPV<br>**Rolling average over 3 months*	DHIS2	Similar to the indicator above, this takes a 3-month rolling average of IPV doses administered to assess if the number is relatively stable from month to month, or if there are large upward/downward variations. It serves as another tracer vaccine.

The tool has established target ranges for each of these ratios/indicators to help interpret the data. For example, for the ratio of PCV1 doses administered to Penta1 doses administered:

- a "green" score is given to a value of 1,

- a "yellow" score is given for value of 0.9-1.10 (excluding 1), and
- a "red" score is given to values <0.90 or >1.10.

For details on calculations and target ranges for all six indicators, see Annex 1.

The possible actions to take column populates suggested actions based on green/yellow/red scores for the most recent month displayed.

Vaccine coverage table

The woreda dashboard also displays a table with vaccine coverage-related information, including RED Categorization and Penta1-Penta3 dropout (DOR).

When available, coverage is calculated using revised target population estimates as the denominator. Revised target population estimates are those obtained through community census ("head counts"), or may be obtained through a combination of other data sources such as newborn registration systems, immunization campaign data, or others; methods may vary by area, as local areas use their own approaches/contexts to estimate targets.

If revised target population figures are not available for a given woreda, coverage is calculated using a conversion factor calculation for the denominator, similar to figures used in DHIS2.

The data source for the denominator (either revised target population figure or conversion factor) is always indicated at the bottom of the woreda vaccine coverage table.

TIP: What does a negative DOR mean?
Negative DOR from Penta1 to Penta3 is sometimes observed. It can be caused by several things, including: poor data quality, movement of communities, health workers vaccinating children from out of their catchment area, or a mix of these factors. If a woreda has a negative DOR, they should explore the reasons for it.

Part IV: Data review and decision-making

IDTT data review team

A cross-departmental working group should be assembled at regional level to bring together managers in immunization, supply chain, and HMIS programs. This team will lead data analysis and decision-making through the use of the IDTT and regular monthly meetings.

The makeup of these groups may vary between regions, but may include:

Position	Department/Directorate	Remark
Regional EPI Officer	RMNCH	Leads IDTT data review efforts and decision-making for immunization program
Regional HMIS Officer	Planning & Resource	Advises on DHIS2-related issues
Hub Vaccine & Supply Chain Officer	PSA	Advises on supply chain related issues
RMNCH Directorate Director	RMNCH	Provides additional decision-making support and guidance during data review meetings

By creating a cross-functional, collaborative group that has the ability to make decisions on behalf of immunization, supply chain, and HMIS programs at regional level, the IDTT data review team will be able to enable holistic decision-making and instigate action on issues that affect immunization outcomes.

IDTT data review meeting preparation and process

Regional level staff have many responsibilities and competing priorities; not everyone will have the time and ability to download and individually examine triangulated data using the IDTT. As such, triangulated data from the IDTT is best utilized in the context of a data review meeting (see Annex 2 for an example IDTT data review meeting agenda). Analysis and preparation for the IDTT data review meeting will be led by the EPI Officer.

During the meeting, the IDTT data review team can discuss analysis and insights obtained from the tool and decide on a course of action to improve performance, both of the immunization program and the supply chain. Some examples of decisions or actions to take based on the IDTT data review meeting discussion may be: more directed supportive supervision, data quality control approaches, changes in stock management, more directed outreach efforts, or others.

The specific activities required for this triangulated data monthly review process are outlined below:

Task/activity	Responsible	Timing
Download DHIS2 report for previous month and send to EPI Officer	HMIS Officer	2 weeks prior to IDTT review meeting
Download mBrana report for previous month and send to EPI Officer	Vaccine Supply Chain Officer	2 weeks prior to IDTT review meeting
Upload DHIS2 and mBrana reports into IDTT Excel tool	EPI Officer	2 weeks prior to IDTT review meeting

Task/activity	Responsible	Timing
Prepare analysis and initial agenda items to discuss; share tool and any analysis via email w team<br>Note: review action points from last month's meeting to assess progress	EPI Officer	Share analysis and agenda items at least 3 days before the meeting
Initial review/comments and flag additional agenda items to discuss in meeting	IDTT data review team	1-3 days before the meeting
Hold monthly meeting (focus on identified agenda items)	IDTT data review team; RMNCH Directorate Director as chair	
Follow up on action points and provide feedback to respective woredas based on IDTT review meeting discussion	Varies, TBD based on action item/program	Begin by 1 week after meeting

From analysis to action

As described, the tool provides suggested actions to take depending on the data/results. Discussing as a team during data review is critical to facilitate decision-making and prioritize actions based on the data.

Follow-up actions may lie with different departments. For example, you may have an issue w stock balances/stockouts in woreda A, an issue with tracking immunization defaulters in woreda B, and an issue with submitting DHIS2 data timely in woreda C; this may require follow-up support from the Vaccine & Supply Chain Officer, the EPI Officer, and the HMIS Officer, respectively.

Working as a cross-departmental team, you can:
- decide as a group how to prioritize follow-up action,
- divide responsibility for follow-up actions, and
- work together to address challenges more holistically.

The Annex 3 template for data review meeting notes can help guide discussion during the IDTT data review meeting and focus follow-up action items. Progress on the action items can then be shared back during the next IDTT data review meeting.

Annex 1: Definitions of DHIS2 and mBrana triangulated indicators

Six woreda-level indicators:

Indicator	Calculation	Target ranges for determining score of indicator
Ratio of PCV1 doses administered to Penta1 doses administered	# doses administered PCV1 / # doses administered Penta1	**Green**: value is 1.0<br>**Yellow**: 0.9–1 or 1–1.1<br>**Red**: <0.90 or >1.10
Ratio of total Penta administered to Penta issued<br>**Rolling average over 6 months*	# doses administered Penta (1-2-3) / # doses issued Penta (1-2-3)<br>*(Rolling average over 6 months)*	**Green**: 0.95 – 1.0<br>**Yellow**: 0.8 – 0.95<br>**Red**: <0.8 or >1.0
Ratio of total measles doses opened to total doses issued<br>**Rolling average over 6 months*	# measles doses opened / # measles doses issued<br>*(Rolling average over 6 months)*	**Green**: 0.95 – 1.05<br>**Yellow**: 0.8–.95 or 1.05–1.20<br>**Red**: <0.8 or >1.20
Ratio of ending stock balance for Penta to buffer stock<br>**Compares end stock balance to buffer stock required*	Penta quantity in stock / Penta buffer stock	**Green**: 0.95 – 1.05<br>**Yellow**: 0.75–0.95 or 1.05–1.15<br>**Red**: <0.75 or >1.15
Doses administered for measles<br>**Rolling average over 3 months*	Comparison of doses administered in the month to the average of the previous 3 months	**Green**: doses administered is within 20% of previous three months' average<br>**Red**: doses administered in the month is greater than or less than 20% of the previous three months' average.
Doses administered for IPV<br>**Rolling average over 3 months*	Comparison of doses administered in the month to the average of the previous 3 months	**Green**: doses administered is within 20% of previous three months' average<br>**Red**: doses administered in the month is greater than or less than 20% of the previous three months' average.

Annex 2: Suggested data review meeting agenda

IDTT Data Review Meeting Agenda

Date:
Participants:
Timekeeper/note taker:

**Item**	**Time**	**Facilitator**
Introductions/greetings	5 min	
Update/report-out on progress of previous meeting's action items	15 min	
IDTT analysis and discussion (focus on pre-identified agenda items)	60 min	
Next steps/confirm action items	10 min	
**Total**	**90 min**	

Supplement to: Almiñana A, Bayeh A, Girma D, et al. Early lessons from Ethiopia in establishing a data triangulation process to analyze immunization program and supply data for decision making. Glob Health Sci Pract. 2022;10(3):e2100719. https://doi.org/10.9745/GHSP-D-21-00719

Annex 3: Suggested template for data review meeting notes

IDTT Data Review Meeting Notes

Date:
Participants:
Timekeeper/note taker:
Reviewing data up through month:

**Reference (IDTT tab/indicator name)**	**Team comments**	**Action item**

Supplement to: Almiñana A, Bayeh A, Girma D, et al. Early lessons from Ethiopia in establishing a data triangulation process to analyze immunization program and supply data for decision making. Glob Health Sci Pract. 2022;10(3):e2100719. https://doi.org/10.9745/GHSP-D-21-00719
